# Periprosthetic Joint Infection: Clinical and Bench

**DOI:** 10.1155/2013/134786

**Published:** 2013-12-17

**Authors:** Mel S. Lee, Andrew Freiberg, Wolfgang Klauser, Christopher S. Mow, Shin-Yoon Kim

**Affiliations:** ^1^Department of Orthopaedic Surgery, Chang Gung Memorial Hospital, Chang Gung University, Linkou, Kweishan 333, Taiwan; ^2^Department of Orthopaedic Surgery, Massachusetts General Hospital, Boston, MA 02114, USA; ^3^HELIOS ENDO-Klinik Hamburg, Holstenstraße 2, Hamburg 22767, Germany; ^4^Department of Orthopaedic Surgery, Stanford University Medical Center, Palo Alto, CA 94304, USA; ^5^Skeletal Diseases Genome Research Center, Department of Orthopaedic Surgery, Kyungpook National University, Graduate School of Medicine, Daegu 702-701, Republic of Korea

Periprosthetic joint infection (PJI) is a devastating complication for the patient and the health care providers. Its incidence is between 1% and 3% in primary and 4% and 6% in revision total joint arthroplasties. The diagnosis can be straightforward with purulent discharge from the joint but may also be confusing especially when associated with medical morbidities. Often, infection leads to multiple operations, prolonged use of antibiotics, extensive utilization of medical resources, and substantial social, economic, or even psychological impacts on the patients, family, hospitals, physicians, and payers. It is estimated that the direct medical cost for treating a PJI is 3 times to the medical cost without infection in revisions and 10 times to the medical cost with uneventful primary cases. As the demand for total joint arthroplasties and the burden of PJI increase globally, knowledge and technologies for detecting, preventing, and managing PJI need to be shared to provide better care to patients.

In this special issue, we included studies of economic analysis on the treatment of PJI (D. Hernandez-Vaquero et al.), animal models of implant-associated infection (M. Haenle et al. and A. I. Stavrakis et al.), methods to improve the diagnosis (D. S. Evangelopoulos et al. and M. S. Lee et al.), efficacy and the potential use of preformed antibiotic-loaded cement spacer (D. Regis et al. and D. W. Chen et al.), and clinical studies of using antibiotic-loaded cement spacer in hip and knee infections (S. S. Ahmad et al. and K. Uchiyama et al.). The notion to publish the special issue is to bridge the basic studies with the clinical studies. Because PJI is a great mimic that many failed joint arthroplasties initially attributed to aseptic loosening are found to be caused by infection in many occasions. M. Haenle et al. and A. I. Stavrakis et al. highlighted the importance of biofilm formation and the possibility of implant-associated infection with comparatively low dose of bacterial inocula. Sonication of the retrieved prosthesis (D. S. Evangelopoulos et al.) and the use of molecular probes for bacteria-specific genes (M. S. Lee et al.) can improve the accuracy of diagnosis. It therefore can help differentiating easy-to-treat or difficult-to-treat cases (S. S. Ahmad et al.) and the choice of antibiotics in fabricating the cement spacers.

 This special issue cannot cover all the issues dealing with PJI. However, by increasing the clinicians and surgeons awareness about PJI, the burdens and challenges could be met by putting more efforts to prevent infection, to design novel bacteria-resistant implants, to improve diagnostic sensitivity and accuracy, to make better antibiotic-loaded spacers, and to develop strategies in treating drug-resistant strains or fungal infections. These works need the collaboration between academic researchers and clinicians to translate basic science to clinical practice.

*Mel S. Lee* *Mel S. Lee* *Andrew Freiberg* *Andrew Freiberg* *Wolfgang Klauser* *Wolfgang Klauser* *Christopher S. Mow* *Christopher S. Mow* *Shin-Yoon Kim* *Shin-Yoon Kim*

